# Fecal metagenomic profiling in patients with colorectal adenomas to characterize gut microbial composition and functional potential

**DOI:** 10.3389/fmicb.2026.1842365

**Published:** 2026-07-08

**Authors:** Guo Zhili, Liu Jie, Xue Yuyue, Yu Fang, Ren Dianqun, Zhang Qin, Lou Xiaojun

**Affiliations:** 1 Department of Oncology, Jiaxing Hospital of Traditional Chinese Medicine, Jiaxing University, Jiaxing, Zhejiang, China; 2 Department of Oncology, Putuo Hospital Affiliated to Shanghai University of Traditional Chinese Medicine, Shanghai, China; 3 Department of Pathology, Jiaxing Hospital of Traditional Chinese Medicine, Jiaxing University, Jiaxing, Zhejiang, China; 4 Department of Gastroenterology, Jiaxing Hospital of Traditional Chinese Medicine, Jiaxing University, Jiaxing, Zhejiang, China

**Keywords:** colorectal adenoma, colorectal cancer, colorectal carcinoma, gut microbiota, metagenomic sequencing

## Abstract

**Objective:**

To investigate differences in gut microbiota between patients with colorectal adenoma (CRA) and healthy individuals using metagenomic sequencing, and to analyze the correlation between microbial abundance and polyp diameter and number.

**Methods:**

Metagenomic sequencing was performed on fecal samples from 60 patients with CRA and 30 healthy controls. Species-level and functional analyses of the gut microbiome were conducted.

**Results:**

Metagenomic profiling revealed a distinct microbial signature in CRA. Statistical analysis identified significant differences in taxonomic composition between the two groups. Overall, 487 genes showed significant abundance differences. Among these, approximately 55.37% were significantly enriched in the adenoma group, suggesting specificity for CRA, while 175 genes were significantly reduced. Alpha diversity analysis indicated similar microbial richness and evenness between the groups, whereas beta diversity confirmed significant structural differences in the microbial community. KEGG enrichment analysis of the top 20 differentially abundant species showed that these microbes were primarily associated with metabolic pathways. The greater number of increased versus decreased genes implied a more pronounced expansion of pathogenic bacteria relative to the loss of beneficial bacteria. Linear discriminant analysis effect size (LEfSe) analysis indicated that *Fusobacterium nucleatum*, Alistipes, and *Bacteroides fragilis* could serve as diagnostic microbial biomarkers for CRA. LEfSe further identified 38 differentially abundant bacterial clades, with genera such as Bacteroides, Peptostreptococcus, and Parabacteroides enriched in patients. Finally, correlation analysis linked the abundance of specific microbial taxa with polyp number and diameter.

**Conclusion:**

This study confirms distinct gut microbiota profiles in patients with CRA compared with healthy individuals, highlights significant microbiome alterations associated with CRA, and reveals novel correlations between specific microorganisms and polyp characteristics, suggesting that microbial changes may contribute to adenoma development.

## 1 Introduction

Colorectal carcinoma (CRC) is one of the three most commonly diagnosed cancers globally and a leading malignancy of the digestive tract, ranking third in incidence and second in mortality worldwide (Sung et al., 2021). Although the mortality rate for CRC remains high, early detection and active intervention are associated with a relatively favorable overall survival. The genetic alterations in CRC typically accumulate over many years, often beginning with the loss of the tumor suppressor gene adenomatous polyposis coli (APC), followed by potential activating mutations in genes such as KRAS and PIK3CA, and the inactivation of TP53 (Zhang et al., 2023). While most CRC cases are sporadic, they frequently develop from dysplastic adenomas through a multi-step progression known as the adenoma-carcinoma sequence (Coker et al., 2022).

CRC is among the diseases most closely linked to the gut microbiota, and research in this area is extensive (Song et al., 2024; Sullivan et al., 2022). Traditional approaches often employ broad-spectrum antibiotic cocktails to deplete the microbiota when investigating pathogenic mechanisms; however, these methods fail to identify the specific microbial species and genes involved (Chen et al., 2022). Notably, Fusobacterium is detected at significantly higher rates in CRC tissues compared to normal colon mucosa and is also enriched in adenomas. The periodontal pathogen *Fusobacterium nucleatum* has been shown to promote myeloid cell infiltration in intestinal tumors in APC (Min/+) mice and is associated with elevated expression of pro-inflammatory genes (e.g., PTGS2/COX-2, SCYB1/IL-8, IL-6, TNF-*α*, and MMP3) in both murine and human studies (Liang et al., 2020). As a critical environmental factor influencing human health, often regarded as our “second genome,” the gut microbiota may integrate other risk factors such as diet, smoking, and age to generate a coordinated signal driving colorectal carcinogenesis, though the mechanisms underlying this process remain to be fully elucidated (Gao et al., 2010).

The gut ecosystem harbors a diverse microbiota that coordinates numerous host physiological and pathological processes. It plays key roles in metabolism, nutrition, immunity, and defense, and is intimately linked to intestinal disorders (Cong et al., 2022). Based on their effects on the host, gut microbes are commonly categorized as beneficial, harmful, or neutral (Wang et al., 2021). The microbiota is actively involved throughout the adenoma-carcinoma sequence (Sheng et al., 2022), and distinct microbial profiles have been reported among healthy individuals, patients with colorectal adenoma (CRA), and those with CRC. For instance, Wang et al. (2022) observed increased abundance and enrichment of *Fusobacterium nucleatum* in patients with CRA. Kordahi et al. (2021) indicated a close association between CRA and *Bacteroides fragilis*. Dejea et al. (2018) suggested that colibactin and *Bacteroides fragilis* toxin may be linked to the transition from CRA to CRC. Furthermore, Lu et al. (2016) found *Pseudomonas mediterranea* to be enriched in patients with CRA and closely associated with carcinogenesis.

In this study, we analyzed metagenomic sequencing data from fecal samples of 60 patients with CRA and 30 healthy controls (HC). Fecal samples reasonably reflect the distal gut environment and can also provide insights into proximal colonic conditions, including early malignant signs. Our aim was to reveal potential correlations between various risk factors, particularly age, sex, polyp number, diameter, and type, and gut microbial alterations during the CRA sequence.

## 2 Materials and methods

### 2.1 Research design

All participants were recruited from patients who underwent colonoscopy or individuals undergoing physical examinations at Jiaxing Hospital of Traditional Chinese Medicine Affiliated to Zhejiang Chinese Medical University from June 2023 to December 2023. Overall, 60 fecal samples from patients with CRA and 30 from healthy individuals (all aged between 18 and 70 years) were collected for this study. The healthy individuals and patients with CRA were grouped into the HC and the CRA groups, respectively. Patient inclusion criteria comprised meeting the diagnostic criteria for CRA. Patients diagnosed with CRAwere enrolled in this study. Fecal samples were collected from these 60 patients with CRA and 30 HC for gut microbiome analysis. Inclusion criteria were: (1) age 18–70 years; (2) patients with CRA confirmed by colonoscopy and pathology. For the HC group, no abnormalities were found on colonoscopy. Exclusion criteria included: (1) familial colorectal cancer or familial adenomatous polyposis; (2) use of antibiotics or probiotics within the past 3 months; (3) presence of infectious symptoms within the past week; and (4) presence of other intestinal diseases. Additional exclusions were pathologically confirmed malignant lesions or highly suspected malignancy, active intestinal inflammation, history of colorectal bleeding or surgery, colonoscopy performed within the past 6 months, lactation, pregnancy or planning pregnancy, residence in the local area for < 3 months, and intake of probiotics within 1 month.

A questionnaire using case report forms was administered to all participants, covering age, sex, surgical history, height, weight, dietary habits, past medical history, medication history, and history of smoking and alcohol consumption. All participants in this study underwent routine bowel preparation. Colonoscopies were performed by experienced gastroenterologists. This study was approved by the Ethics Review Committee of Jiaxing Hospital of Traditional Chinese Medicine (Ethics No.: Jia Zhong Zhong Lun Shen No. 2024–02021). This study was also conducted in accordance with the Declaration of Helsinki.

### 2.2 Sample collection

Fecal samples from all participants (qualified stool sample collected before eating between 5:00 a.m. and 8:00 a.m. before bowel preparation medication for colonoscopy) were immediately collected into 10 mL sterile centrifuge tubes after defecation. They were temporarily transferred to a − 20 °C freezer and subsequently stored long-term at −80 °C until DNA extraction. Furthermore, all fecal samples were processed within 6 months and transported to LC Bio Technology CO., Ltd. (Hangzhou, China) for metagenomic sequencing.

### 2.3 Metagenomic sequencing materials and methods

#### 2.3.1 Experimental methods

Total DNA was extracted from all samples using a magnetic bead-based kit, quantified with Qubit, and fragmented by ultrasonication to 200–500 bp. Libraries were constructed using the TruSeq Nano DNA LT Kit, involving end repair, A-tailing, adapter ligation, and PCR amplification, then sequenced on the Illumina NovaSeq 6,000 platform in PE150 mode. Raw reads were filtered for quality and adapter contamination, and host sequences were removed where applicable. Clean reads were assembled per sample using *de novo* assembly, and CDS were predicted from contigs ≥500 bp using MetaGeneMark. Redundant sequences were clustered at 95% identity and 90% coverage using CD-HIT to generate a non-redundant unigene set. Bowtie2 was used to map clean reads to unigenes, and unigenes with total counts ≤2 across all samples were discarded. Abundance of each unigene was calculated based on mapped read counts and gene length. Taxonomic annotation was performed against the NR_meta database, and functional annotation against nine databases (GO, KEGG, eggNOG, CAZy, CARD, PHI, MGEs, VFDB, BacMet). Diversity, differential abundance, and enrichment analyses were subsequently conducted at multiple levels.

### 2.4 Data analysis

The Kruskal-Wallis test was used for the comparison between the two groups. The inter-group differences in the Euclidean distance of metabolites and the Bray-Curtis distance of bacteria were tested. All statistical analyses were conducted using R programming (version 3.6.1) for data preprocessing, statistical analysis, and model construction. To control for baseline confounding, we used propensity score matching (PSM) to perform 1:2 nearest neighbor matching between the CRA group and the control group. The propensity score was estimated through logistic regression, and covariates included age, gender, BMI, and history of hypertension. The cut-off value was set as 0.2 times the standard deviation of the propensity score. After matching, the standardized mean difference (SMD) was used to evaluate the balance, and an SMD < 0.1 was considered as balance. The matched samples were used for subsequent metagenomic difference analysis and correlation analysis, and all analyses were based on the post-matching dataset.

## 3 Results

### 3.1 Clinical baseline data table

**Figure 1:**
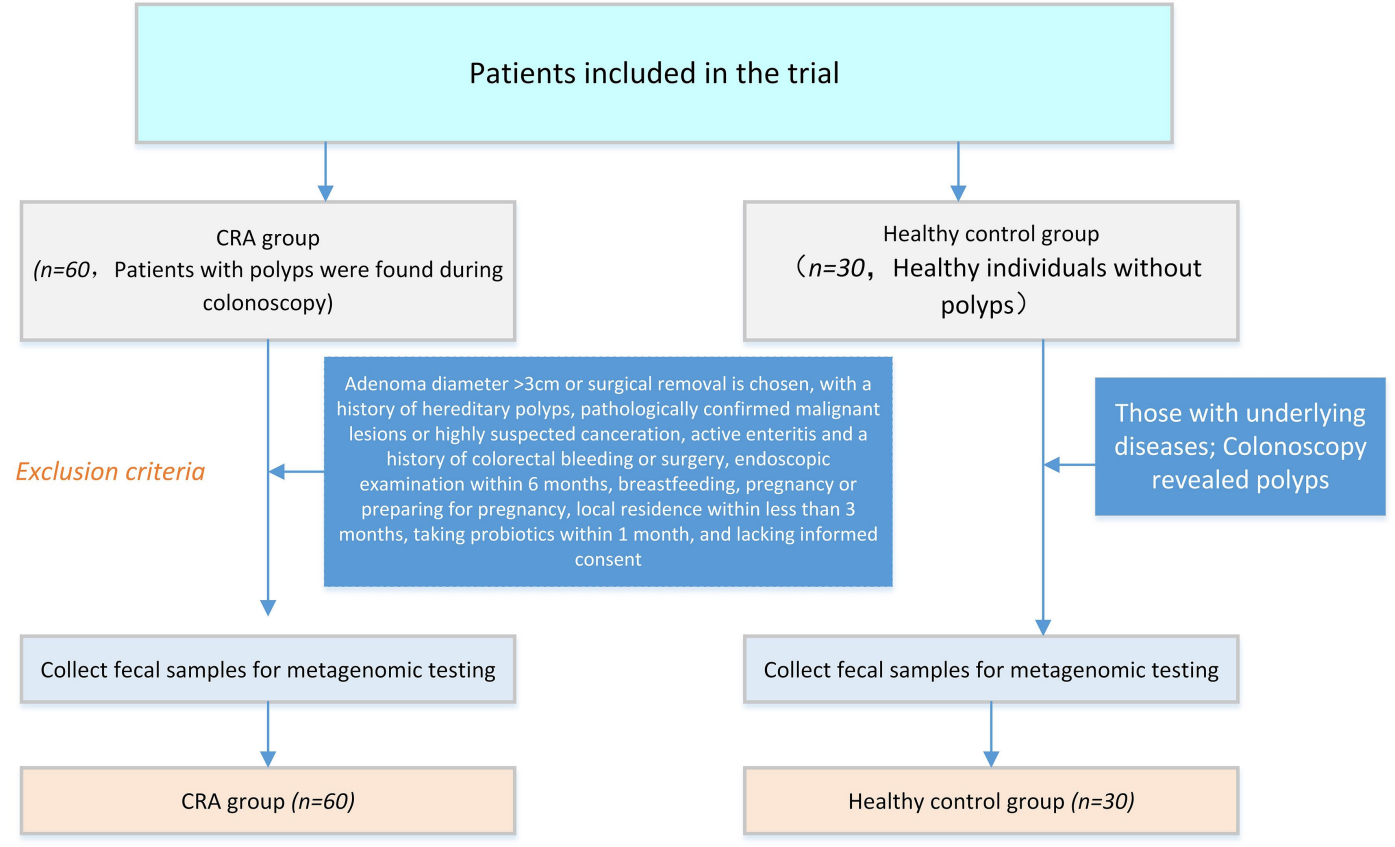
Flowchart showing the case included in the study.

The age of the CRA group was significantly higher than that of the HC group (*p* < 0.001), and the difference was statistically significant. The BMI in the CRA group was higher than that in the HC group (*p* = 0.041). The proportion of males was higher in the CRA group (*p* = 0.025), and this difference was statistically significant. The proportion of individuals with a history of hypertension was higher in the CRA group compared with the HC group (*p* = 0.001), and the difference was statistically significant (Figure 1).

Given that there were baseline differences between the CRA group and the HC group in terms of age, sex, BMI, and history of hypertension (Supplementary Table 1), all comparisons between the groups were conducted using multivariate adjustment models to control for these potential confounding effects. Specifically, when assessing the beta diversity of the microbiota (PERMANOVA), and when using MaAsLin2/DESeq2 to identify different species/pathways, age, sex, BMI, and history of hypertension were all included as covariates in the model. Additionally, as a sensitivity analysis, we performed propensity score matching (1:2 nearest neighbor matching) to obtain a baseline-balanced subgroup, and repeated the core analysis within this subgroup to verify the robustness of the main findings (Table 1).

**Table 1:** Comparison of baseline characteristics between the CRA group and the healthy control group before and after propensity score matching.

Baseline data	HC group (*n* = 27)	CRA group (*n* = 54)	SMD	*p*
Age (At the age of)	52.11 ± 10.86	53.04 ± 11.29	0.084	0.721
BMI, M (Q₁, Q₃)	21.90 (19.13, 24.04)	23.33 (22.04, 25.39)	0.072	0.041
Sex, *n* (%)			0.091	0.025
Female	21 (70.00)	27 (45.00)		
Male	9 (30.00)	33 (55.00)		
History of hypertension, *n* (%)			0.042	0.045
No	30 (100.00)	43 (71.67)		
Yes	0 (0.00)	17 (28.33)		
History of diabetes mellitus, *n* (%)			0.069	0.179
No	30 (100.00)	54 (90.00)		
Yes	0 (0.00)	6 (10.00)		
History of coronary heart disease, *n* (%)			0.049	0.533
No	30 (100.00)	57 (95.00)		
Yes	0 (0.00)	3 (5.00)		

*p* < 0.05 was statistically significant.

Continuous variables are presented as mean ± standard deviation or median (interquartile range); categorical variables are presented as number (percentage). The pre-matching *p*-values are derived from an independent sample t-test, Mann–Whitney U test or chi-square test; the post-matching *p*-values are derived from a paired sample test or McNemar test. PSM adopts nearest neighbor 1:2 matching with a clamping value of 0.02.

### 3.2 Differences in the abundance of intestinal microbiota species between the two groups

**Figure 2:**
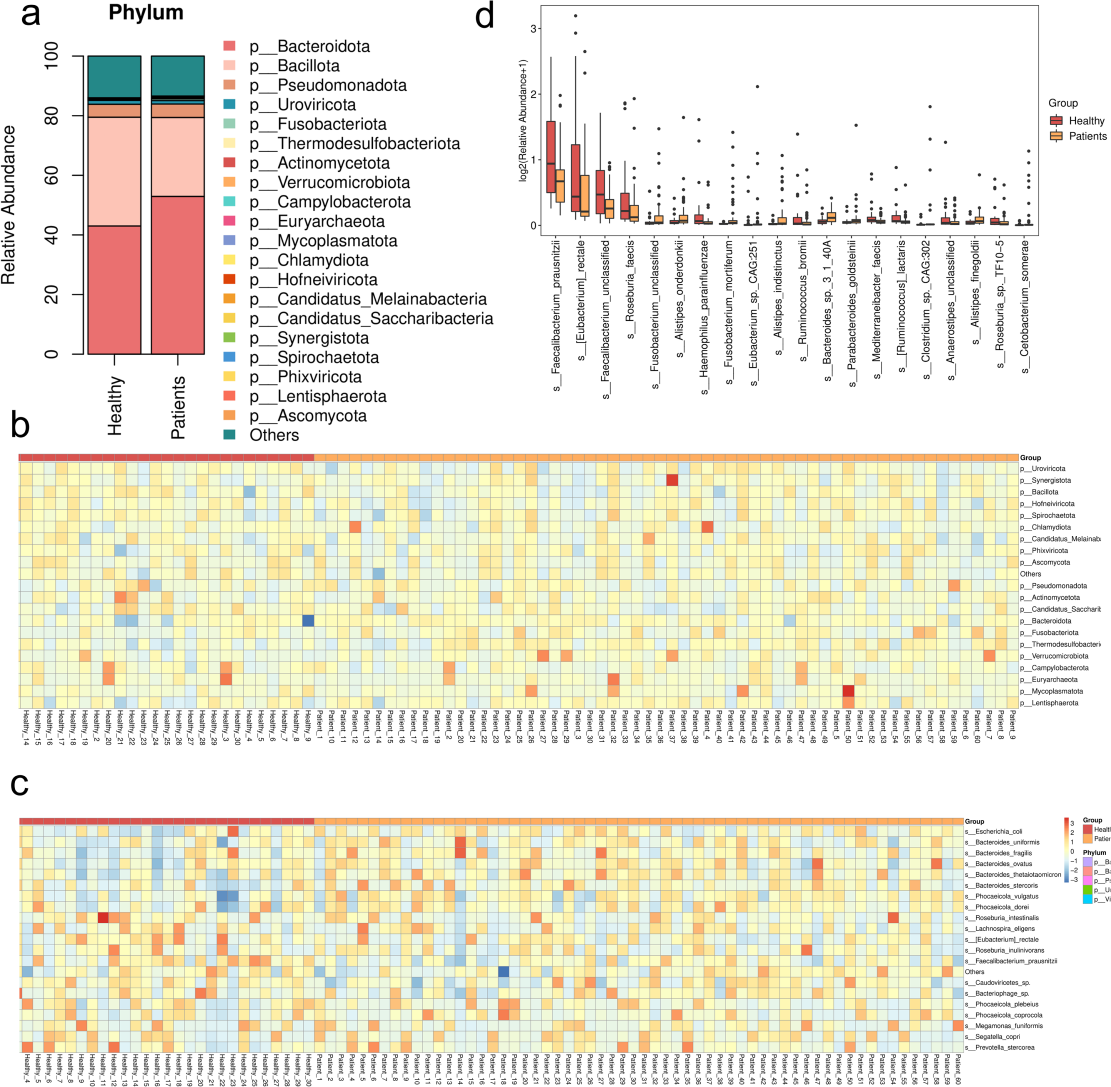
**(a)** Column stacked bar plot of species abundance at the phylum level (the abscissa represents the sample name, and the ordinate represents the species abundance). **(b)** Heat map of species abundance at the phylum level. **(c)** Heat map of species abundance at the species level **(d)** Box plot of different species (the horizontal coordinate represents the top 20 species with significant differences (marked “yes”), ordered from the highest to lowest abundance from left to right). The ordinate represents log2-transformed relative abundance (relative abundance + 1). The box plot consists of five parts: maximum, upper quartile (Q3), median, lower quartile (Q1), and minimum. Black points above and below the box are outliers.

We collected fecal samples from the above-mentioned cases and conducted species detection of the fecal genomes to examine the differences in genetic species. To obtain the abundance information of species at different taxonomic levels in various samples, we integrated the species annotation information with the unigene abundance data. The abundance at a specific taxonomic level equals the sum of the abundances of all unigenes annotated to that level. The stacked bar chart of species abundance at the phylum level indicated that Bacteroidota, Bacillota, and Pseudomonadota were the predominant bacterial groups in the gut (Figure 2a). The heatmap of species abundance at the phylum level (Figure 2b) showed that compared with the HC group, the CRA group had significantly increased abundances of Mycoplasmatota, Verrucomicrobiota, Chlamydiota, and Lentisphaerota, while the abundance of Actinomycetota was significantly decreased. The heatmap of species abundance at the species level (Figure 2c) revealed that, compared with the HC group, the relative abundance of *Bacteroides fragilis* was significantly increased in the CRA group, whereas the relative abundances of Phocaeicola plebeius and *Megamonas funiformis* were significantly decreased. Statistical analysis of differences was performed for the top 20 most abundant bacteria at the species level (Figure 2d; Table 2). The results showed that compared to the HC group, the CRA group had significantly decreased relative abundances of *Faecalibacterium prausnitzii*, *Eubacterium rectale*, and *Roseburia faecis*, while the relative abundances of *Fusobacterium nucleatum* and Alistipes were significantly increased.

**Table 2:** Statistical analysis of the differences at the species level among the top 20 bacteria with the highest abundance.

Sequence number	Unigene_ID	Mean (overall)	Mean (patients)	Mean (healthy)	Log2FC	Regulation	*p* value	Q value	Significance
1	s__Selenomonas_sp._oral_taxon_136	0.0010435056	0.0000206579	0.0030892011	−7.22	down	0	0	yes
2	s__Saccharomyces_cerevisiae	0.0001062507	0.0000758195	0.0001671133	−1.14	down	0	0	yes
3	s__Methanogenium_sp._S4BF	0.0000653640	0.0000001338	0.0001958245	−10.52	down	0	0	yes
4	s__Siphoviridae_sp._ctVFN1	0.0000207526	0.0000000627	0.0000621325	−9.95	down	0	0	yes
5	s__Podoviridae_sp._cthJQ11	0.0001484156	0.0000005127	0.0004442216	−9.76	down	0.00	0.00	yes
6	s__Desulfovibrio_sp._86	0.0001147172	0.0001685583	0.0000070352	4.58	up	0.00	0.00	yes
7	s__Veillonella_sp._KGMB01456	0.0000971377	0.0001264952	0.0000384228	1.72	up	0.00	0.00	yes
8	s__Cloacibacillus_sp._An23	0.0028144568	0.0040924064	0.0002585577	3.98	up	0.00	0.00	yes
9	s__Bacteroidales_bacterium_WCE2004	0.0002137821	0.0002980324	0.0000452815	2.72	up	0.00	0.00	yes
10	s__Siphoviridae_sp._ctOXk3	0.0001672720	0.0002509080	0.0000000000	Inf	up	0.00	0.00	yes
11	s__Halomonas_heilongjiangensis	0.0000868331	0.0001217686	0.0000169621	2.84	up	0.00	0.00	yes
12	s__Fibrobacter_sp._UWCM	0.0000849336	0.0001257422	0.0000033163	5.24	up	0.00	0.00	yes
13	s__Siphoviridae_sp._ctprd3	0.0003928327	0.0005892491	0.0000000000	Inf	up	0.00	0.00	yes
14	s__Mosigvirus_mar005p1	0.0000699266	0.0000001021	0.0002095757	−11	down	0.00	0.00	yes
15	s__Agromyces_flavus	0.0000517504	0.0000754187	0.0000044139	4.09	up	0.00	0.01	yes
16	s__Alistipes_sp._An66	0.0027542757	0.0038560840	0.0005506591	2.81	up	0.00	0.01	yes
17	s__Porphyromonas_gulae	0.0003796331	0.0005270463	0.0000848066	2.64	up	0.00	0.01	yes
18	s__Fundicoccus_ignavus	0.0049179661	0.0000513980	0.0146511024	−8.16	down	0.00	0.01	yes
19	s__Haemophilus_sputorum	0.0039821171	0.0006204341	0.0107054830	−4.11	down	0.00	0.01	yes
20	s__Myoviridae_sp._ct3Sw5	0.0002698555	0.0003419259	0.0001257148	1.44	up	0.00	0.01	yes

### 3.3 Analysis of the diversity of gut microbiota between healthy individuals and patients with CRA

**Figure 3:**
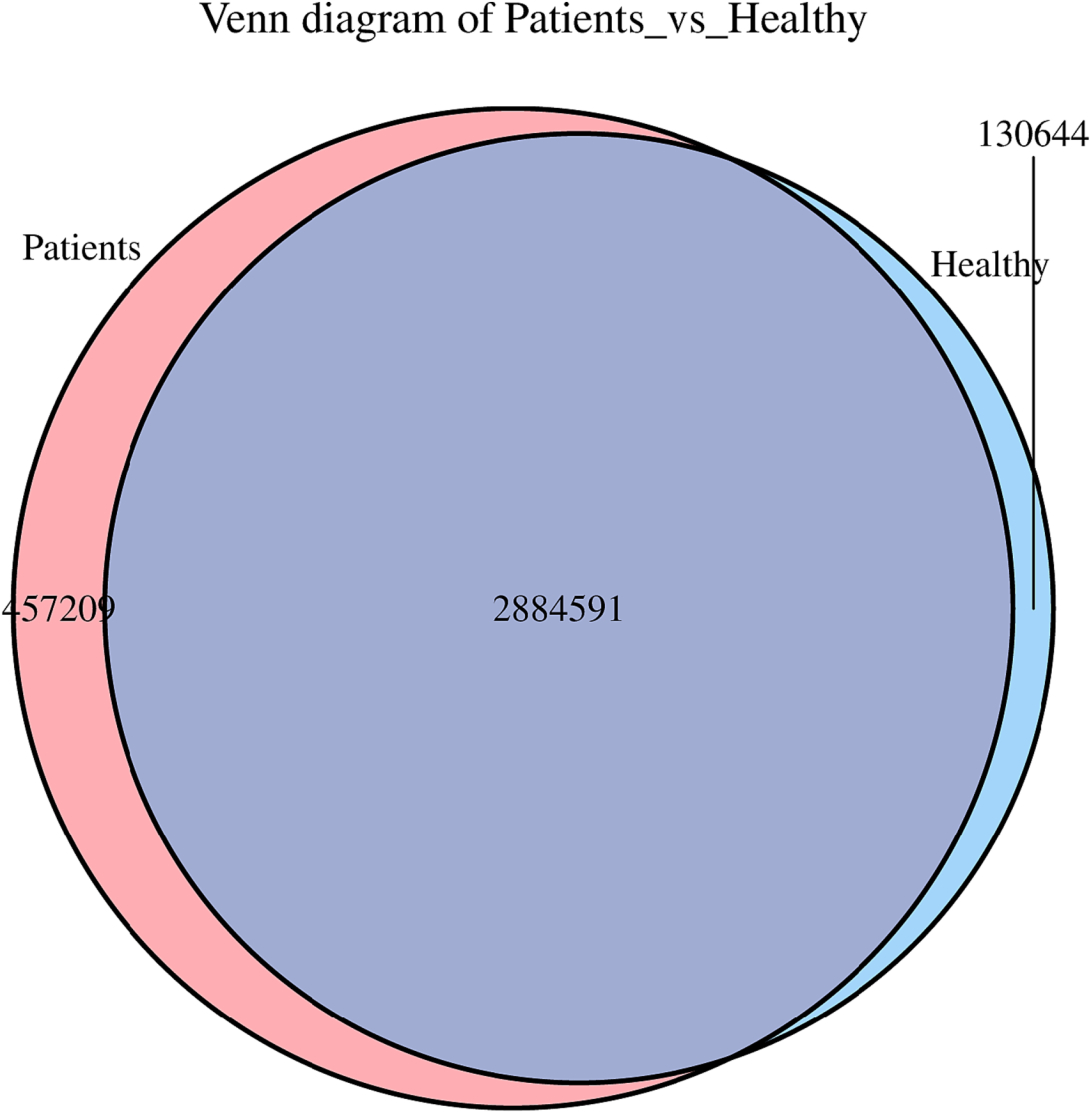
Venn diagram of unigene composition in the gut microbiota. The Venn diagram shows the number of shared and unique unigenes between the CRA group and the HC group. The gut microbiota contained 2,884,591 unigenes across the two groups, while the CRA group and HC group had 457,209 and 130,644 unigenes, respectively.

Based on the species abundance table, we performed differential species testing between sample groups. The screening threshold for differential species was set at *p* < 0.05 and |log2(fold_change)| > 1. Venn diagrams are used to count the number of unigenes shared and unique to multiple sample groups, providing an intuitive representation of the similarity and specificity of unigene composition across environmental samples. The Venn diagram shows the number of unigenes shared and unique to the CRA and HC groups (Figure 3). The two groups shared 2,884,591 unigenes, while the CRA and HC groups had 457,209 and 130,644 unique unigenes, respectively,the filtered effective genes amount to 32,489. Among these, 487 unigenes showed significant differences, with 312being up-regulated and175down-regulated.

**Figure 4:**
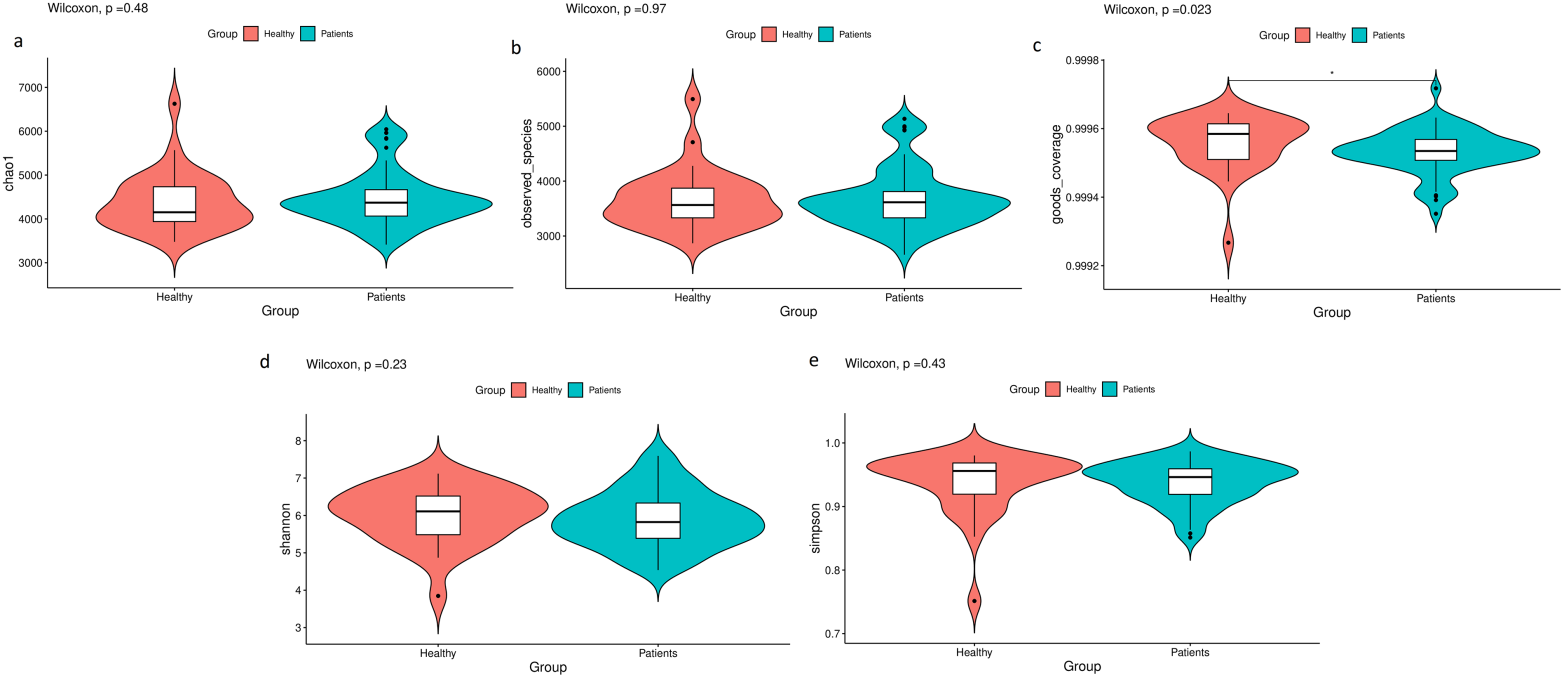
Alpha diversity of gut microbiota diversity by **(a)** Chao1, **(b)** Observed_species, **(c)** Good’s coverage, **(d)** Shannon, and **(e)** Simpson indices. *p*-values for overall differences between groups were obtained using the Wilcoxon test.

Alpha diversity analysis (primarily using indices such as Chao1, Observed_species, Good’s coverage, Shannon, and Simpson) was employed to assess the richness and evenness of the gut microbiota in the two groups (Figure 4). Alpha diversity refers to the diversity within a specific environment or ecosystem and is mainly used to reflect species richness, evenness, and sequencing depth. It is primarily evaluated using indices such as Chao1, Observed_species, Good’s coverage, Shannon, and Simpson, which indicate richness and evenness. The Good’s coverage index showed a significant difference between the two groups (*p* < 0.05). No statistically significant differences were observed in the Chao1, Observed_species, Shannon, and Simpson indices between the two groups (*p* > 0.05). This indicates that the richness of the microbial community composition was similar between the fecal samples of patients with CRA and normal healthy individuals, without significant differences. The alpha diversity assessed by the Shannon index showed a decreasing trend in gut microbial diversity in patients with CRA, but this trend did not reach statistical significance.

**Figure 5:**
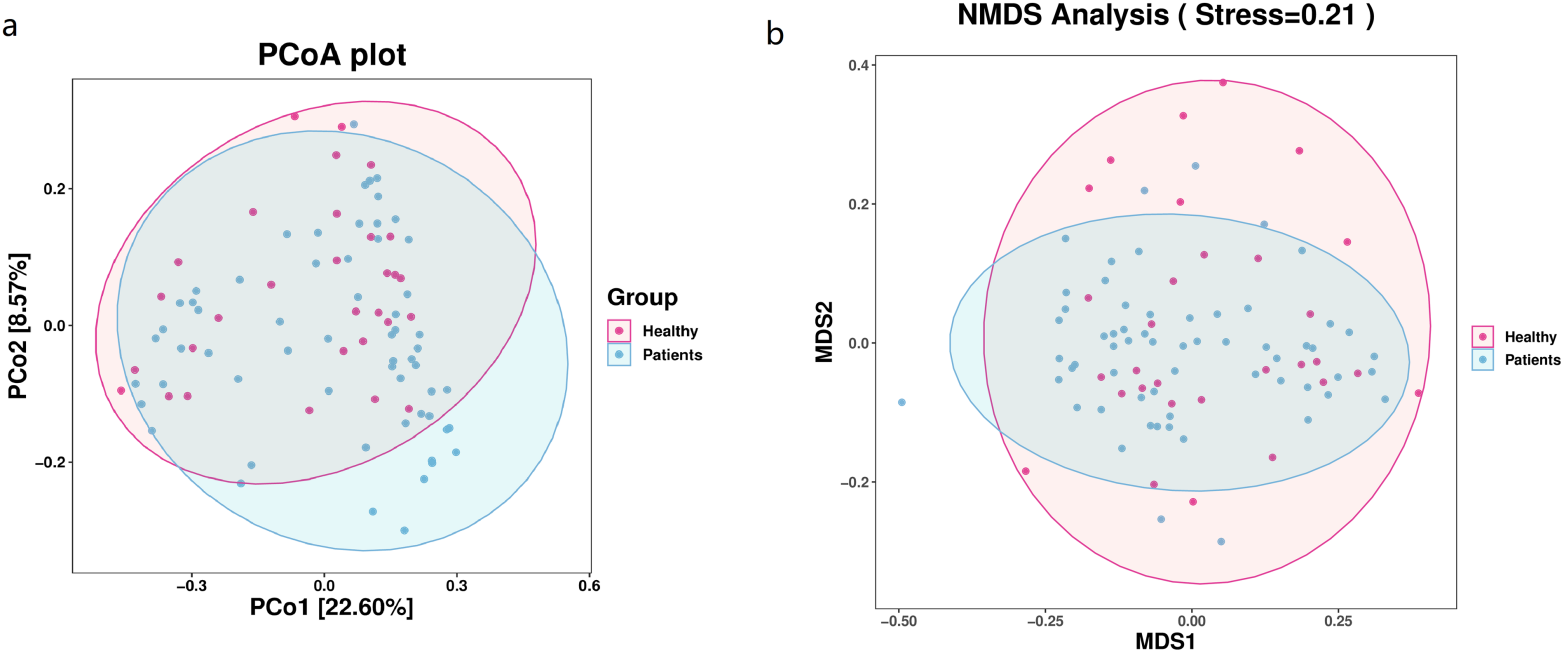
**(a)** Principal coordinate analysis (PCoA) of gut microbiota based on Bray–Curtis distance. PCo1 and PCo2 explain 22.60 and 8.57% of the total variance, respectively. The PERMANOVA test shows significant differences between groups (*p* = 0.002). A clear separation is observed in the microbial composition between the patient group and the healthy group, suggesting that the disease state may be related to the structure of the microbial community. **(b)** NMDS analysis diagram. MDS1 (horizontal axis) and MDS2 (vertical axis) form a two-dimensional ordination space used to intuitively reflect the similarity or difference between samples. Stress = 0.21 is at the edge of acceptability, indicating that this two-dimensional graph can generally reflect the true relationship between the samples, and the sample points of the healthy group and the patient group in the sorting space show a clear separation trend, suggesting that there are overall differences in the intestinal microbiota structure between the two groups.

Beta diversity refers to the species compositional differences between different environmental communities. Together with alpha diversity, it constitutes the overall diversity or biological heterogeneity of a given set of environmental communities. PCoA based on Bray-Curtis distance was performed to visualize the differences between the two groups (Figure 5a). The distance between samples is represented by two principal coordinates (PCo1 and PCo2), where samples closer together are more similar in composition. The Adonis test yielded a significant result (*p* = 0.01), indicating a significant difference in gut microbiota structure between the two groups. Beta diversity analysis based on the NMDS method also showed a significant difference in microbial community composition between patients with CRA and HC (*p* < 0.01) (Figure 5b). The differences in gut microbial composition between patients with CRA and HC were primarily observed in the four dominant phyla: Firmicutes, Bacteroidota, Fusobacteriota, and Pseudomonadota. Compared to the HC group, patients with CRA showed significantly increased relative abundances of Bacteroidota and Fusobacteriota, while the relative abundance of Firmicutes was significantly decreased (*p* < 0.05). In patients with colorectal cancer, 30 taxa, including the genera Bacteroides, Peptostreptococcus, and Parabacteroides, and the family Porphyromonadaceae, were significantly enriched. In contrast, the HC group was predominantly characterized by the family Streptospirillaceae and the genus Blautia, showing eight significantly enriched taxa. The results of the Wilcoxon rank-sum test further validated the abundance differences at the genus level: the relative abundances of Alistipes, Bacteroides, and Parabacteroides were all significantly higher in patients with colorectal cancer than in healthy individuals (all *p* values < 0.05), with the differences for Bacteroides and Parabacteroides being highly statistically significant.

### 3.4 Functional enrichment analysis of differential microbiota

**Figure 6:**
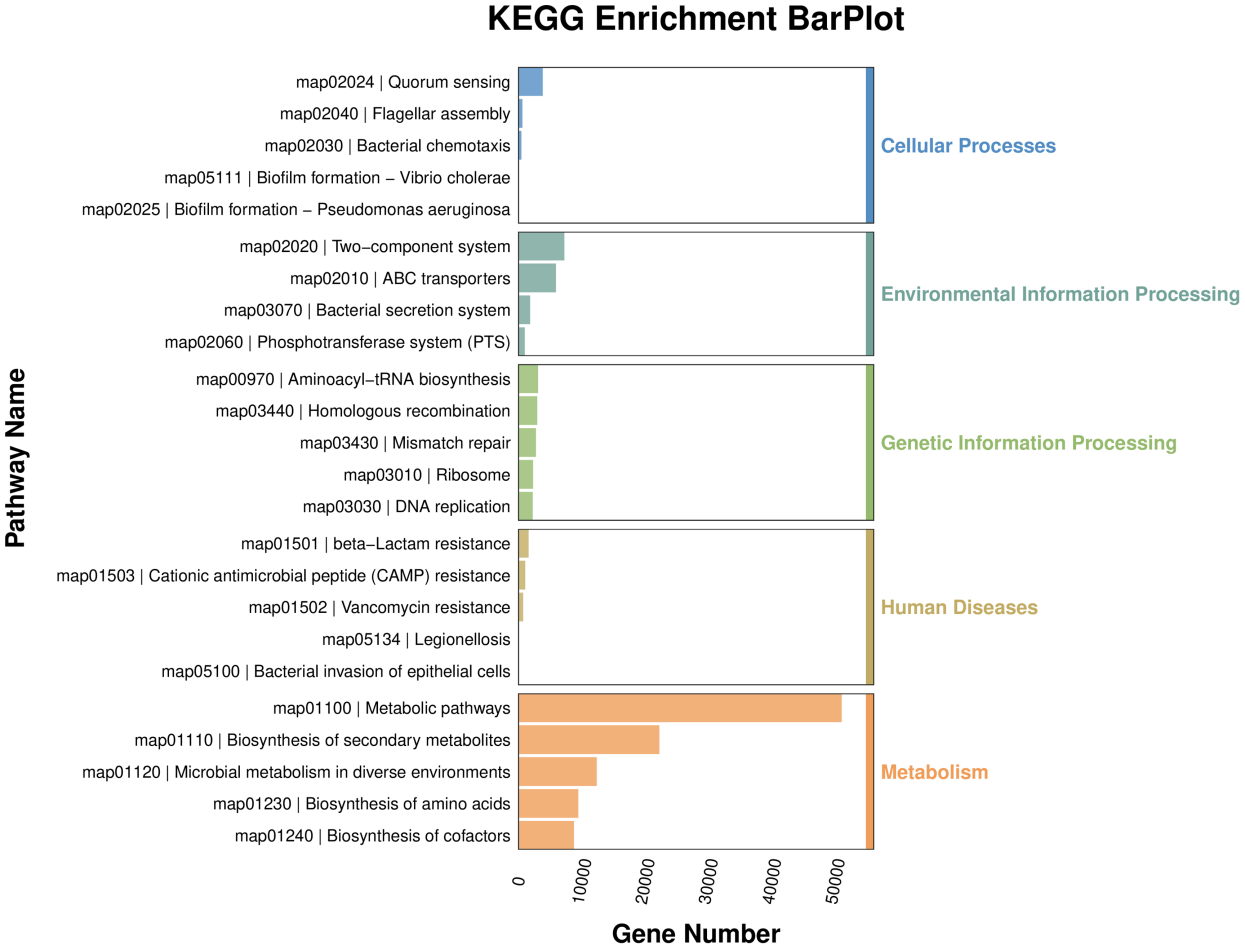
Functional annotation map of the two groups of differential flora. In the figure, horizontal columns represent different samples or groups, vertical columns represent the relative abundance ratio of each functional item, different colors represent different functional items, the height of the same column color represents the height of the same functional item in different samples/groups, and the higher the column, the higher the proportion. Function items other than the top 20 in abundance are classified as “Others”.

**Figure 7:**
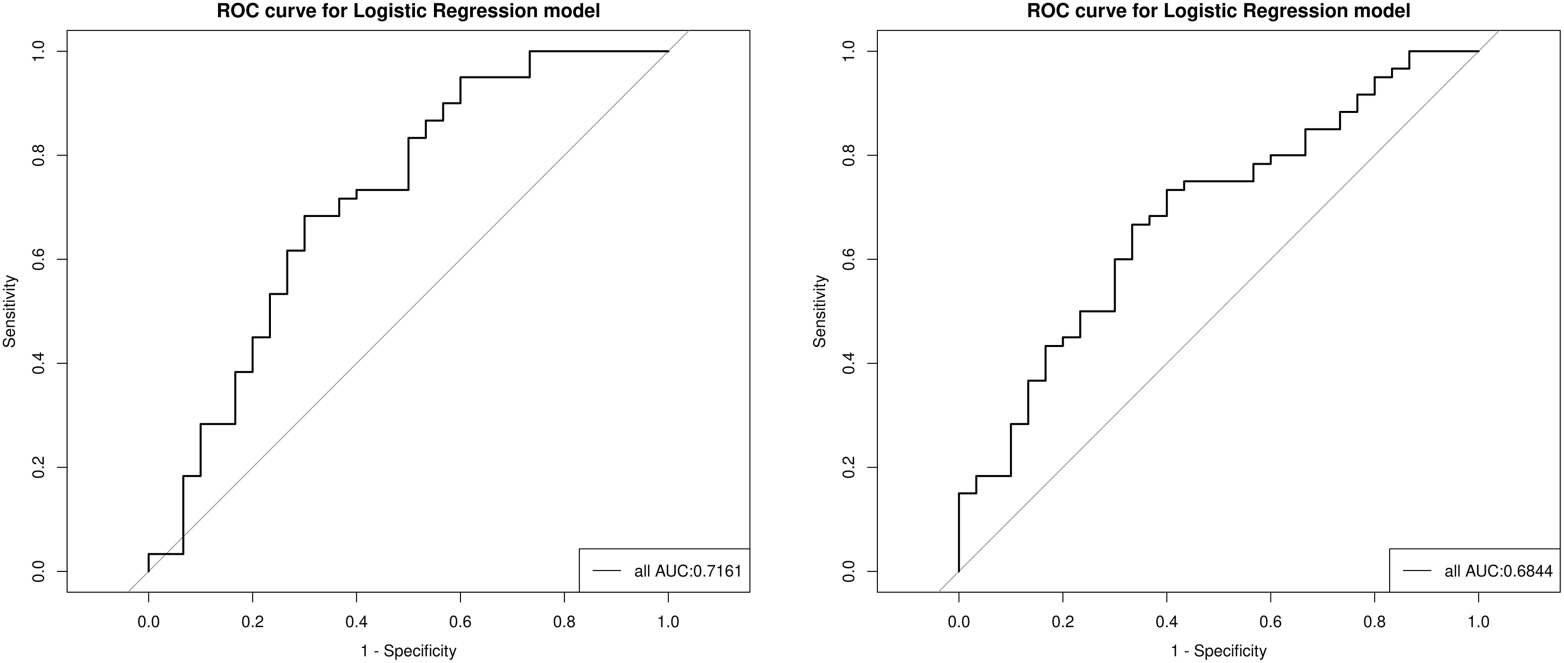
ROC curve of the Logistic regression model for determining the abundance of gut microbiota at species and genus levels for distinguishing colorectal adenoma from healthy controls. **(a)** Species-level model AUC = 0.7161; **(b)** Genus-level model AUC = 0.6844; The species-level microbiota characteristic model has a higher discriminatory efficacy.

In order to further analyze the relationship between species-level genes that cause the differences and the occurrence of adenomas, we conducted a further bioinformatics analysis of these genes. Functional annotation of the differential microbiota was performed using the KEGG database, while genes performing the same functions were clustered together. The results showed that a large number of genes were enriched in metabolic categories mainly including metabolism, (Figure 6), indicating that the microbiota plays an important role in these metabolic processes. Further functional enrichment analysis at the metabolic level revealed that metabolic pathways, biosynthesis of secondary metabolites, microbial metabolism in diverse environments, biosynthesis of amino acids, and biosynthesis of cofactors showed significant differences between the two groups. We also constructed a Logistic regression model at the species and genus levels to analyze the abundance of intestinal microbiota in colorectal adenomas and healthy controls (Figure 7). The ROC curve indicated that the model based on species-level microbiota characteristics had a higher discriminatory efficiency.

Based on the aforementioned KEGG pathway enrichment analysis, we systematically investigated the potential functional relevance of these pathways to the initiation and progression of CRA. Biofilm formation serves as a critical adaptive strategy for bacterial colonization, functioning as a protective “microbial fortress” that enhances bacterial adherence to the intestinal epithelium and confers resistance against mechanical clearance by peristalsis, host immune surveillance, and antimicrobial agents (Wolf et al., 2022). Notably, significant enrichment of biofilm-associated pathways, including those specific to *Pseudomonas aeruginosa* and Vibrio cholera, suggests that opportunistic pathogens may establish persistent, spatially organized communities on the CRA mucosal surface, thereby delivering sustained pro-inflammatory and proliferative stimuli to the underlying epithelium (Liu et al., 2025). Such functional capabilities facilitate stable microbial colonization at adenoma-prone sites and foster a localized, chronic source of microbial-driven epithelial perturbation. Concurrently, pronounced enrichment of pathways governing DNA replication, DNA repair (including homologous recombination and mismatch repair), and ribosomal biogenesis indicates that the associated bacterial community exhibits robust proliferative activity, high-fidelity genomic maintenance, and efficient protein synthesis, traits that collectively support rapid population expansion and competitive dominance within the dynamic intestinal niche. Furthermore, the gut microbiota contributes to tumorigenesis by remodeling the local metabolic microenvironment, a process termed “oncometabolic reprogramming” (Zhili et al., 2025). Key mechanisms include, direct modulation of host epithelial signaling, e.g., polyamines and secondary bile acids act as bioactive metabolites that activate oncogenic pathways such as Wnt/*β*-catenin; induction of epithelial DNA damage via genotoxic metabolites, including hydrogen sulfide and colibactin (a genotoxin whose biosynthesis is linked to secondary metabolic pathways); degradation of the protective mucus layer by metabolites such as hydrogen sulfide, resulting in compromised epithelial barrier integrity and increased exposure to microbial antigens and toxins; and immunomodulation, specific microbial metabolites (e.g., certain short-chain fatty acids and tryptophan derivatives) can suppress antitumor immunity by altering the phenotype and function of local immune cells, including regulatory T cells and macrophages (Yan et al., 2026).

### 3.5 LEfSe analysis of the intestinal microbiota in two groups

**Figure 8:**
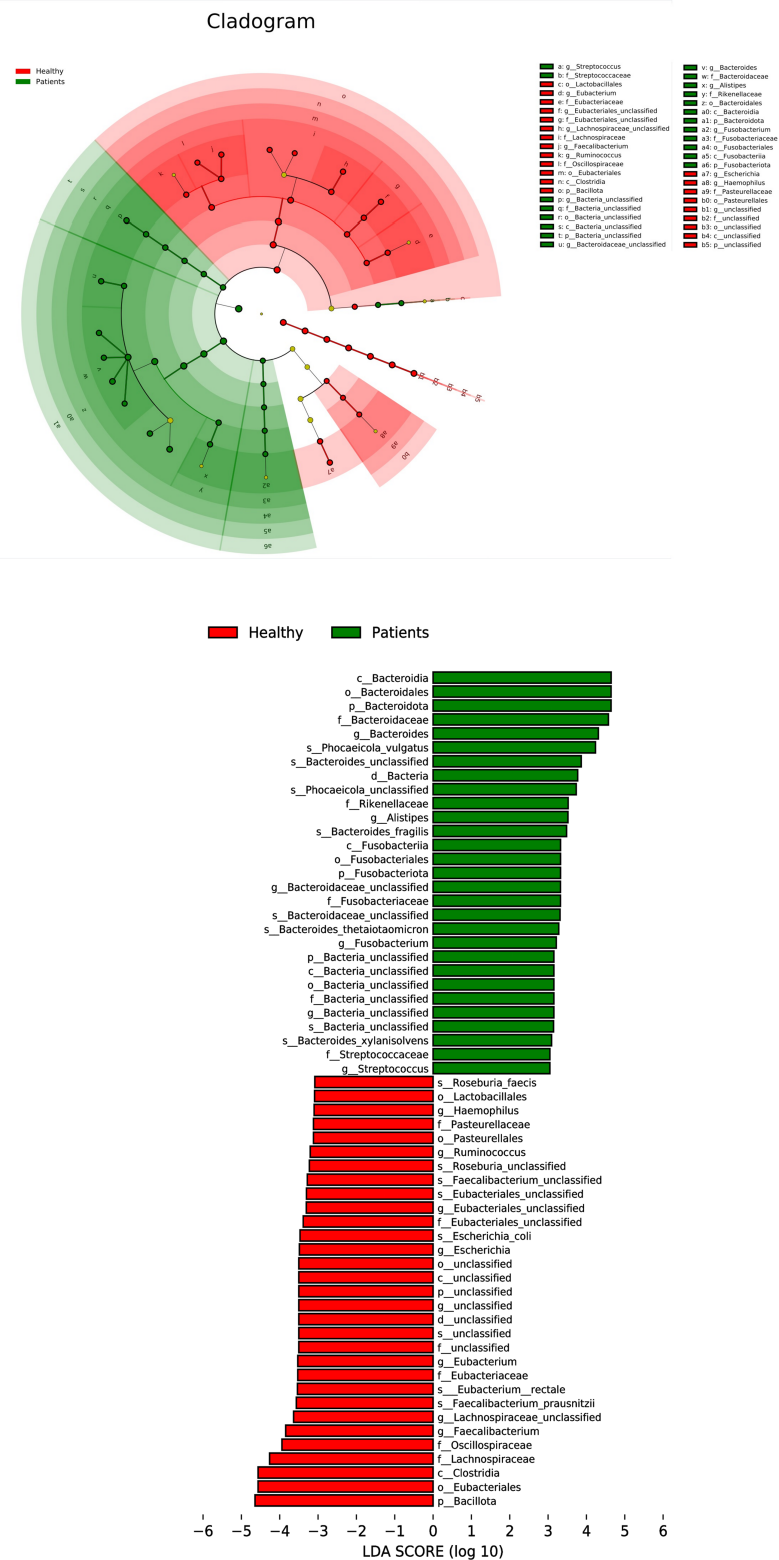
**(a)** Evolutionary branch diagram (different circles show the evolutionary relationship among the seven taxonomic levels: kingdom, phylum, class, order, family, genus and species from inside to outside). Individual nodes represent taxa at each level, and larger node sizes indicate a higher relative abundance of the corresponding taxon. Nodes colored yellow indicate no significant difference from the HC group, while red shows a significant difference. Taxa with significant differences are directly marked in the figure, and taxa at other levels are identified by letters. **(b)** Distribution bar chart (significantly different taxa with LDA score greater than the preset value, i.e., biomarker with statistical significance, the default threshold is 3.0). The color of the bar chart indicates the group in which the abundance of each different taxon is higher, and the bar length represents the LDA score, i.e., the degree of influence of significantly different species between groups.

Finally, we used LEfSe to select the species with significant differences. LEfSe analysis is primarily employed to compare two or more groups and identify microbial taxa that exhibit significant differences in abundance across these groups, serving as potential biomarkers. In this study, LEfSe analyses were conducted across seven taxonomic levels for predefined comparison groups (Figures 8a,b), with a significance threshold set at LDA score > 3.0 and *p*-value < 0.05. Differential taxa were visualized using cladograms and bar charts depicting their relative abundances. The analysis revealed distinct microbial profiles between the HC and CRA groups. Specifically, the CRA group was enriched with Lactobacillales, Haemophilus, Pasteurellaceae, Pasteurellales, Ruminococcus. In contrast, the HC group showed higher abundances of Bacteroidia, Bacteroidales, Bacteroidota_f, Bacteroidaceae_g, Bacteroides, Phocaeicola, and *Bacteroides vulgatus*. These differentially abundant taxa warrant further investigation due to their potential biological and functional relevance.

### 3.6 Correlation analysis of the two groups of intestinal microbiota with indicators such as the patient’s age and the number of intestinal polyp diameters

**Figure 9:**
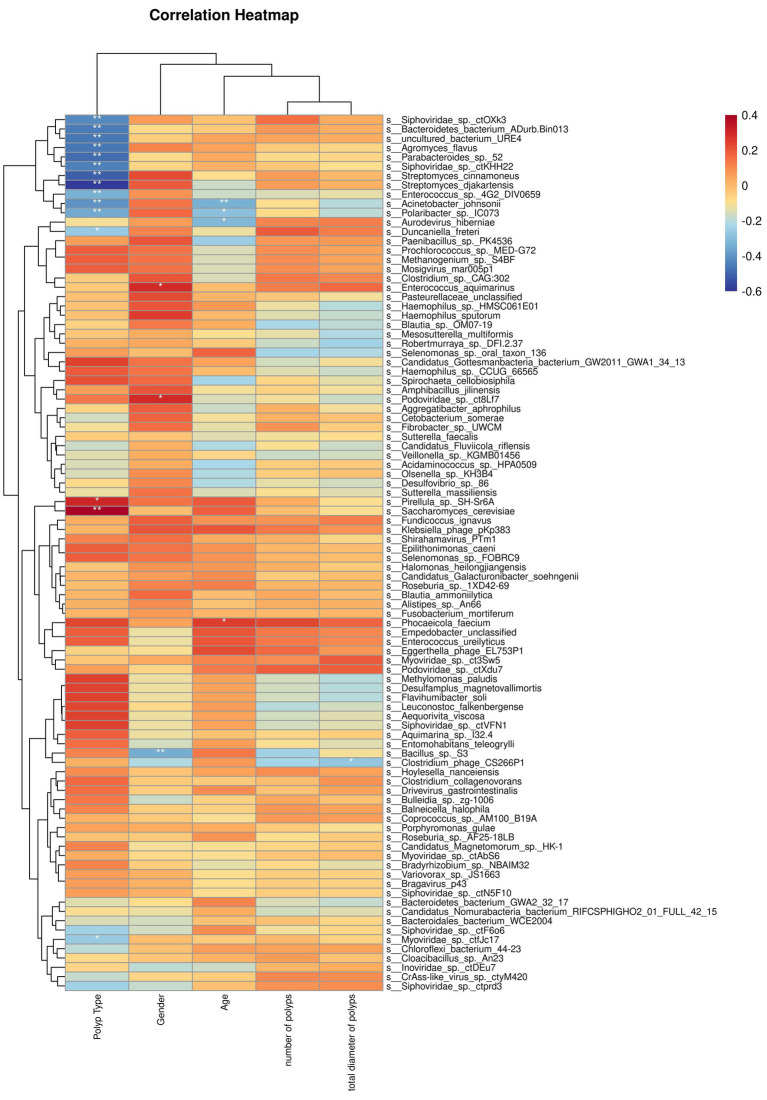
Heat map of correlation analysis. Used to present various types of microorganisms (different species marked on the right) and “polyp type,” “sex,” “age,” “number of polyps,” “total diameter of polyps,” showing the Spearman correlation coefficient and the significance of the correlation among these phenotypic characteristics. Red represents positive correlation, and the darker the color, the stronger the positive correlation. Blue represents negative correlation, and the darker the color, the stronger the negative correlation. Labels such as * and ** are used to reflect the significance level of the correlation. The more labels there are, the more statistically significant the correlation is. As can be seen from the figure, there are differences in the correlations between different microorganisms and various phenotypic characteristics. Some microorganisms show a darker red color with “polyp type,” indicating that there is a strong positive correlation between the relative abundance of these microorganisms and the polyp type. It is possible that the abundance of this type of microorganisms is higher in the samples of the corresponding type of polyps. Some microorganisms show a darker blue color with “total diameter of polyps,” meaning that the larger the total diameter of polyps, the lower the abundance of such microorganisms may be, and the negative correlation is relatively strong. Overall, this heat map can visually screen out the microbial groups significantly associated with the polyp-related phenotypes, providing preliminary clues and directions for subsequent research on the relationship between intestinal microbiota and the occurrence and development of polyps.

Through correlation analysis between microbiota data and patients’ clinicopathological features (including polyp size, total diameter, type, age, and gender), we systematically evaluated the impact of these risk factors on gut microbial alterations associated with CRA. The correlation heatmap (Figure 9) intuitively displays the Spearman correlation coefficients and their significance between specific microbial taxa and various clinical phenotypes (polyp type, sex, age, number of polyps, total polyp diameter).

The analysis revealed the following key associations: Polyp type: Prevotella was enriched in hyperplastic polyps, whereas the Bacteroides genus was positively correlated with adenomatous polyps. Sex: The Lactobacillus genus was more enriched in female patients, while certain species within the Ruminococcaceae family were more prominent in males. Age: The abundance of Enterococcus increased with age, while Roseburia exhibited a negative correlation with age. Number of polyps: Streptococcus and *Escherichia coli*-related metabolic lineage groups showed positive correlations with an increased number of polyps, whereas Bifidobacterium showed a negative correlation. Total polyp diameter: *Fusobacterium nucleatum* and *Bacteroides fragilis* were positively correlated with larger polyp diameter. Conversely, the abundance of Prevotella and butyrate-producing bacteria (e.g., Faecalibacterium) decreased as the total diameter increased.

We conducted a multivariate linear/logistic regression analysis on the significant correlations of all reports. In the model, we used microbial abundance as the main predictor variable and included age, sex, BMI, and history of hypertension as covariates to control for the influence of these potential confounding factors (Supplementary Table 2). The positive correlation between Fusobacterium nucleatum and polyp diameter remained robust after adjustment (*β* = 0.32, *p* < 0.001).

## 4 Discussion

This study investigated the gut microbiota in healthy individuals and patients with CRA. Previous research has highlighted the essential roles of gut microbiota in metabolism, nutrition, immunity, and host defense. Moreover, dysbiosis has been closely linked to intestinal diseases such as CRA and CRC, providing a rationale for exploring gut microbes as non-invasive diagnostic and prognostic tools for CRA. Gut dysbiosis frequently drives pathological changes in intestinal disorders (Saito et al., 2019). Microbial imbalance can trigger local intestinal inflammation, and the pro-inflammatory cytokines produced during chronic inflammation further stimulate tumor initiation and progression (Molinaro et al., 2018). The tumor microenvironment, composed of immune cells, fibroblasts, inflammatory cells, extracellular matrix, blood vessels, and nerves, is a critical driver of adenoma carcinogenesis. Inflammatory cytokines can mediate complex pathways, including activation of NF-κB and STAT3 signaling, leukocyte recruitment, and promotion of cell proliferation, angiogenesis, lymphangiogenesis, and tumor invasion (Zhang et al., 2026). Therefore, performing species-level and functional analyses to clarify the distinct roles of gut microbiota in patients with CRA versus healthy individuals is crucial for advancing non-invasive diagnostics for CRA and preventing CRC (Moschen et al., 2016).

**Figure 10:**
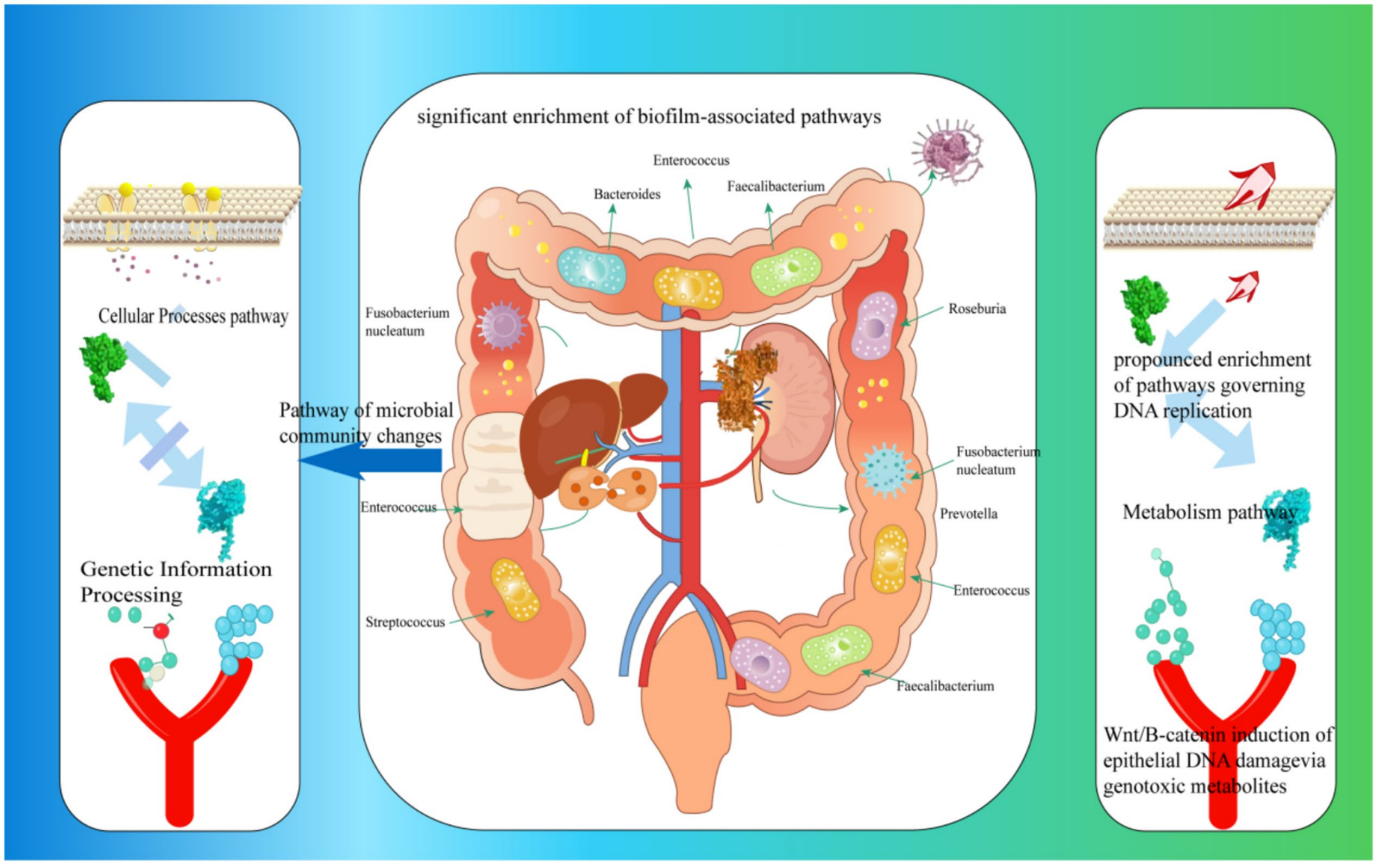
Integrated pathway enrichment analysis of cellular processes, genetic information processing, and metabolism following alterations in gut microbiota. This figure illustrates the key enriched pathways across three major functional categories following alterations in gut microbiotas. In Cellular processes, significant enrichment is observed in biofilm-associated pathways. Genetic information processing highlights the role of Wnt/*β*-catenin signaling in inducing epithelial DNA damage, likely mediated by genotoxic metabolites. In metabolic pathways, pronounced enrichment is observed in pathways governing DNA replication. Together, these findings underscore the interplay between metabolic activity, genetic instability, and cellular adaptive mechanisms.

Based on metagenomic sequencing, alpha-diversity analysis in this study revealed similar microbial richness between patients with CRA and HC group, with no statistically significant differences. This does not imply identical microbial types or quantities, but rather suggests that certain specific microbial groups may be present at comparable abundance levels in both groups, a result that may reflect the inherent complexity of the human gut microbiota and similarities in factors such as diet and lifestyle among individuals. In contrast, beta-diversity demonstrated clear separation between groups, and Bray–Curtis distances indicated significant differences in bacterial community structure. Differential abundance analysis further confirmed substantial taxonomic and compositional differences in the gut microbiota between healthy individuals and patients with CRA (Figure 10).

KEGG functional enrichment analysis showed that differentially abundant microbes were primarily associated with metabolic processes, including general metabolic pathways, biosynthesis of secondary metabolites, microbial metabolism in diverse environments, amino acid biosynthesis, and cofactor biosynthesis. These results indicate substantial enrichment of microbial genes in metabolic categories, underscoring the important role of the microbiota in these biochemical processes. LEfSe analysis across seven taxonomic levels revealed enrichment of *Roseburia faecis*, Lactobacillales, Haemophilus, Pasteurellaceae, Pasteurellales, Ruminococcus, and Roseburia in the CRA group, whereas the HC group showed enrichment of Bacteroidia, Bacteroidales, Bacteroidota, Bacteroidaceae, Bacteroides, Phocaeicola, and *Bacteroides vulgatus*. These differentially abundant microorganisms warrant focused attention (Hasan et al., 2022).

Further correlation analysis between microbiota data and patients’ pathological outcomes (polyp size, total diameter, type), age, and sex revealed the following associations: Prevotella was enriched in hyperplastic polyps, while the genus Bacteroides showed a positive correlation with adenomatous polyps. Lactobacillus was enriched in females, whereas certain species within the family Ruminococcaceae were more prominent in males. The abundance of Enterococcus increased with age, whereas Roseburia exhibited a negative correlation with age. Regarding polyp number, Streptococcus and *Escherichia coli*–related taxa showed positive correlations with higher polyp counts, while Bifidobacterium showed a negative correlation. For total polyp diameter, *Fusobacterium nucleatum* and *Bacteroides fragilis* were positively correlated with larger diameters, whereas the abundance of Prevotella and butyrate-producing bacteria (e.g., Faecalibacterium) decreased as diameter increased. These correlations systematically demonstrate the close relationship between the microbial community and key clinical features of colorectal polyps, providing an ecological basis for investigating the role of the microbiota in precancerous lesions.

Prevotella is a genus capable of degrading complex carbohydrates, but under certain conditions it may also promote local inflammatory responses (Tett et al., 2021). Although hyperplastic polyps have traditionally been considered non-neoplastic, recent studies link them to the serrated adenoma pathway, which also carries malignant potential. The enrichment of Prevotella might foster a microenvironment conducive to mucosal cell hyperproliferation (i.e., “hyperplasia”) via its metabolites (such as succinate) or by inducing low-grade chronic inflammation (Kovatcheva-Datchary et al., 2015). Adenomatous polyps and the Bacteroides genus: Certain species within Bacteroides, particularly *Bacteroides fragilis*, can produce toxins that directly damage intestinal epithelial cell DNA or disrupt the mucosal barrier, thereby initiating or promoting adenoma formation (acante et al., 2020). Since adenomas are recognized precancerous lesions of colorectal cancer, the enrichment of Bacteroides may signal the establishment of a more direct “carcinogenic drive” in the ecological niche.

Lactobacillus is a dominant genus in the vaginal microbiota, and its metabolite lactic acid helps maintain a healthy acidic environment. In the gut, Lactobacillus is also considered beneficial, producing lactic acid, maintaining intestinal acidity, and inhibiting pathogens (Scillato et al., 2021). Its relative enrichment in the female gut may be related to systemic hormone levels (e.g., estrogen), which have been shown to influence gut microbiota structure (Santos-Marcos et al., 2023), potentially partly explaining gender-based differences in colorectal cancer incidence. Ruminococcaceae are important dietary fiber degraders, producing short-chain fatty acids such as butyrate. Butyrate serves as the primary energy source for colonic epithelial cells, exhibits anti-inflammatory properties, and helps maintain intestinal barrier health. Their greater prominence in males may reflect gender-based dietary differences (e.g., higher fiber intake), although the association with polyp risk requires analysis at the species level due to functional diversity within this family.

Enterococcus is an opportunistic pathogen that typically causes infection under conditions of impaired immunity (Sangiorgio et al., 2024). With advancing age, immune function gradually declines (immunosenescence), weakening control over potential pathogens and allowing opportunistic bacteria such as Enterococcus to colonize and expand more readily (Cusumano et al., 2024). Decline of Roseburia: Roseburia is another important butyrate producer; a decrease in its abundance is a classic marker of gut dysbiosis. Aging is often accompanied by a reduction in butyrate-producing bacteria, leading to insufficient colonic energy supply, impaired barrier function, and elevated chronic inflammation, thereby creating a “fertile ground” for polyp formation (Kang et al., 2023). Positive correlation of Streptococcus/*Escherichia coli* with polyp number: Both are often considered “fast-growing” taxa that proliferate rapidly when carbohydrate sources are abundant (Bonnet et al., 2014). Certain strains possess pro-inflammatory properties. Their increase may reflect an imbalanced gut ecology that broadly stimulates the intestinal mucosa, potentially leading to synchronous development of polyps at multiple sites. Bifidobacterium is a well-recognized probiotic that inhibits harmful bacteria and maintains gut health by competing for nutrients and adhesion sites, producing antimicrobial compounds, and modulating immune responses. A decrease in its abundance means the gut loses an important “protective force,” allowing pro-polyp bacterial groups to dominate, which may contribute to multiple polyp formation. *Fusobacterium nucleatum* and *Bacteroides fragilis*: These two species are widely regarded as “keystone pathogens” in colorectal cancer (Wang et al., 2025). *Fusobacterium nucleatum* can directly invade colonic cells via its FadA adhesin, activate procarcinogenic signaling pathways (e.g., *β*-catenin), and form pro-tumor bacterial-immune cell aggregates (biofilms), directly driving polyp growth and malignant progression (Nguyen Duy et al., 2024). Enterotoxigenic *Bacteroides fragilis*: As noted earlier, its toxin can cause direct cellular damage. The positive correlation of its abundance with polyp diameter strongly suggests an active role in promoting polyp growth and progression (Zamani et al., 2024). Decrease of Prevotella and Faecalibacterium with Polyp Diameter: Although certain Prevotella species are enriched in hyperplastic polyps, the genus as a whole is functionally diverse, with many members essential for carbohydrate metabolism and healthy mucosal immunity. A general decrease in its abundance may indicate deterioration of the gut environment (Shukla et al., 2025). Faecalibacterium (notably *F. prausnitzii*) is one of the most important butyrate producers in the human gut and exerts potent anti-inflammatory effects, being hailed as a “next-generation probiotic.” As polyps enlarge and cancer risk rises, the protective, anti-inflammatory butyrate environment is disrupted, leading to the loss of beneficial bacteria such as Faecalibacterium. Its decreased abundance is therefore both a consequence of polyp progression and a factor that may further accelerate disease worsening due to its absence (Shi et al., 2025).

In summary, alterations in gut microbiota structure are closely associated with the clinical characteristics of colorectal polyps. A microbial community skewed toward a “pathogenic” profile is typically characterized by the enrichment of pro-inflammatory or opportunistic pathogenic bacteria (such as *Fusobacterium nucleatum*, *Bacteroides fragilis*, and Enterococcus) and a concomitant reduction in protective, anti-inflammatory bacteria, particularly butyrate producers like Faecalibacterium and Roseburia (Gan et al., 2025). This dysbiotic state, through multiple mechanisms including the induction of chronic inflammation, disruption of the mucosal barrier, direct DNA damage, and activation of oncogenic signaling pathways, collectively fosters polyp initiation, growth, and malignant progression. These findings provide a strong scientific rationale for future applications of the gut microbiota as non-invasive biomarkers for polyp risk assessment, as well as for preventing polyp development and recurrence through microbiota-targeted interventions (e.g., probiotics, prebiotics, and dietary modifications) (Wu et al., 2025). This study confirms distinct gut microbiota profiles in patients with CRA compared to healthy individuals, underscores significant microbiome alterations associated with CRA, and reveals novel correlations between specific microorganisms and host polyp features. The present work has certain limitations, primarily due to the limited sample size, which may constrain the generalizability of the gut microbiota findings for both groups. Therefore, future studies with larger cohorts are needed to validate these results, complemented by further molecular investigations to elucidate the specific underlying mechanisms.

## Data Availability

The raw data generated in this study can be found in the Gene Sequence Archive (GSA) for Human (https://ngdc.cncb.ac.cn/gsa-human), accession HRA012317.
