# Supplementary material for: Fecal metagenomic profiling in patients with colorectal adenomas to characterize gut microbial composition and functional potential
Source: Front Microbiol. 2026 Jul 8;17:1842365. doi: 10.3389/fmicb.2026.1842365 (PMC13388898; doi:10.3389/fmicb.2026.1842365)
Supplement: Supplementary file 1 [file Table_1.DOC]

### ****Supplementary Table 1: Correlation Analysis of Key Microbial Characteristics and Polyp Indicators (Comparison after Simple Correlation and Multivariate Adjustment)****

| **Microbial Feature)** | **(Polyp Trait)** | **Simple correlation coefficient (r) / OR** | **Simple correlation P-value** | **Adjusted β coefficient (95% CI) / Adjusted OR (95% CI)** | **Adjusted P value** |
| --- | --- | --- | --- | --- | --- |
| Fusobacterium nucleatum | Total Diameter | r = 0.42 | <0.001 | β = 0.32 (0.15, 0.49) | <0.001 |
| Bacteroides fragilis | Total Diameter | r = 0.38 | 0.001 | β = 0.28 (0.11, 0.45) | 0.002 |
| Faecalibacterium | Total Diameter | r = -0.35 | 0.003 | β = -0.21 (-0.38, -0.04) | 0.016 |
| Prevotella (genus) | Total Diameter | r = -0.30 | 0.010 | β = -0.15 (-0.32, 0.02) | 0.081 |
| Streptococcus (genus) | Polyp Number | r = 0.39 | 0.001 | β = 0.25 (0.08, 0.42) | 0.005 |
| Bifidobacterium (genus) | Polyp Number | r = -0.33 | 0.006 | β = -0.18 (-0.35, -0.01) | 0.041 |
| Enterococcus (genus) | Age | r = 0.48 | <0.001 | β = 0.40 (0.23, 0.57) | <0.001 |
| Roseburia (genus) | Age | r = -0.36 | 0.002 | β = -0.22 (-0.39, 0.05) | 0.012 |
| Bacteroides Enrichment | Adenomatous vs. Hyperplastic | OR = 3.50 | 0.008 | aOR = 2.85 (1.30, 6.25) | 0.009 |
| Prevotella Enrichment | Hyperplastic vs. Adenomatous | OR = 2.90 | 0.022 | aOR = 2.10 (0.85, 5.19) | 0.108 |

Table Note:

Simple correlation analysis: For continuous variables (diameter, quantity, age), Spearman correlation analysis was used, reporting the correlation coefficient (r) and P value. For categorical variables (type of polyp), logistic regression was employed, reporting the odds ratio (OR) and P value.Multivariate adjustment analysis: Multivariate linear regression (for continuous outcomes) or multivariate logistic regression (for categorical outcomes) was used. All models adjusted for the following covariates: age, gender, BMI, history of hypertension. β coefficient: Indicates the average change in the polyp index for each unit increase in microbial abundance after adjusting for covariates. aOR: Adjusted odds ratio. 95% CI: 95% confidence interval. The adjusted P value marked in bold < 0.05 indicates that the association remains statistically significant after controlling for confounding factors. This table shows only a few examples of key microbial characteristics. The complete results can be found in the supplementary dataset.
